# Growth in Height in Childhood and Risk of Coronary Heart Disease in Adult Men and Women

**DOI:** 10.1371/journal.pone.0030476

**Published:** 2012-01-24

**Authors:** Karri Silventoinen, Jennifer L. Baker, Thorkild I. A. Sørensen

**Affiliations:** 1 Population Research Unit, Department of Social Research, University of Helsinki, Helsinki, Finland; 2 Department of Public Health, University of Helsinki, Helsinki, Finland; 3 Institute of Preventive Medicine, Copenhagen University Hospital, Copenhagen, Denmark; New York University School of Medicine, United States of America

## Abstract

**Background:**

Adult height is inversely associated with the risk of coronary heart disease (CHD), but it is still unknown which phase of the human growth period is critical for the formation of this association. We investigated the association between growth in height from 7 to 13 years of age and the risk of CHD in adulthood.

**Methods and Findings:**

The heights of almost all children born 1930 through 1976 who attended school in the Copenhagen municipality (232,063 children) were measured annually from 7 to 13 years of age. Birth weight data were available since 1936. Fatal and non-fatal CHD events were ascertained by register linkage until 2008 (25,214 cases). Hazard ratios (HR) with 95% confidence intervals (CI) were estimated by Cox proportional hazards regression for height z-scores (standard deviation units) and change in height z-scores. Height z-scores were inversely related to the risk of CHD. The association was strongest at 7 years of age (HR = 0.91, CI 0.90–0.92 in boys and 0.88, CI 0.86–0.90 in girls) and steadily weakened thereafter, yet it still remained at 13 years of age (HR = 0.95, CI 0.94–0.97 and 0.91, CI 0.89–0.93, boys and girls respectively). The associations were not modified by birth weight. Independent of the age-specific risk, rapid growth was associated with an increased CHD risk, most pronounced between 9 and 11 years in girls (HR = 1.22, CI 1.14–1.31) and between 11 and 13 years in boys (HR = 1.28, CI 1.22–1.33) per unit increase in z-score. Adjustment for body mass index somewhat strengthened the associations of CHD risk with height and weakened the association with growth.

**Conclusions/Significance:**

Risk of CHD in adulthood is inversely related to height at ages 7 through 13 years, but strongest in the youngest, and, independently hereof, the risk increased by growth velocity.

## Introduction

The association between the physical development of the child and the adult risk of coronary heart disease (CHD) assessed by CHD risk factor profiles as well as by the incidence of CHD in adulthood has long attracted scientific interest [1], but the developmental pathways are complex. Low birth weight [2], [3] and eventual short adult stature [4], which is positively correlated with low birth weight [5], are associated with an increased adult risk of CHD. There is also evidence that catch-up growth in height and weight among those with low birth weight is associated with an increased CHD risk [6]. Further, the early onset of puberty is associated with hypertension [7] and other metabolic risk factors of CHD [8] but also with shorter adult stature [9]. The background of these associations is poorly understood, but it has been suggested that low birth weight and short adult stature are markers of environmental influences in pre- and post-natal life which may further predispose to metabolic abnormalities [10].

The intermediate period between infancy and adolescence, i.e. childhood, has however received little attention. The period of childhood growth and its associations with CHD risk are interesting both from scientific and public health point of views. First, childhood is characterized by slow growth and its associations with CHD risk may be different than the periods of very rapid growth during infancy and puberty. Second, childhood is a unique period of life from a public health point of view because it allows for school-based screening, monitoring and intervention. Thus we investigated how growth in height from 7 to 13 years of age is associated with risk of CHD throughout adulthood, and whether the association is dependent on birth weight in a large cohort of Danish children.

## Materials and Methods

**Figure 1:**
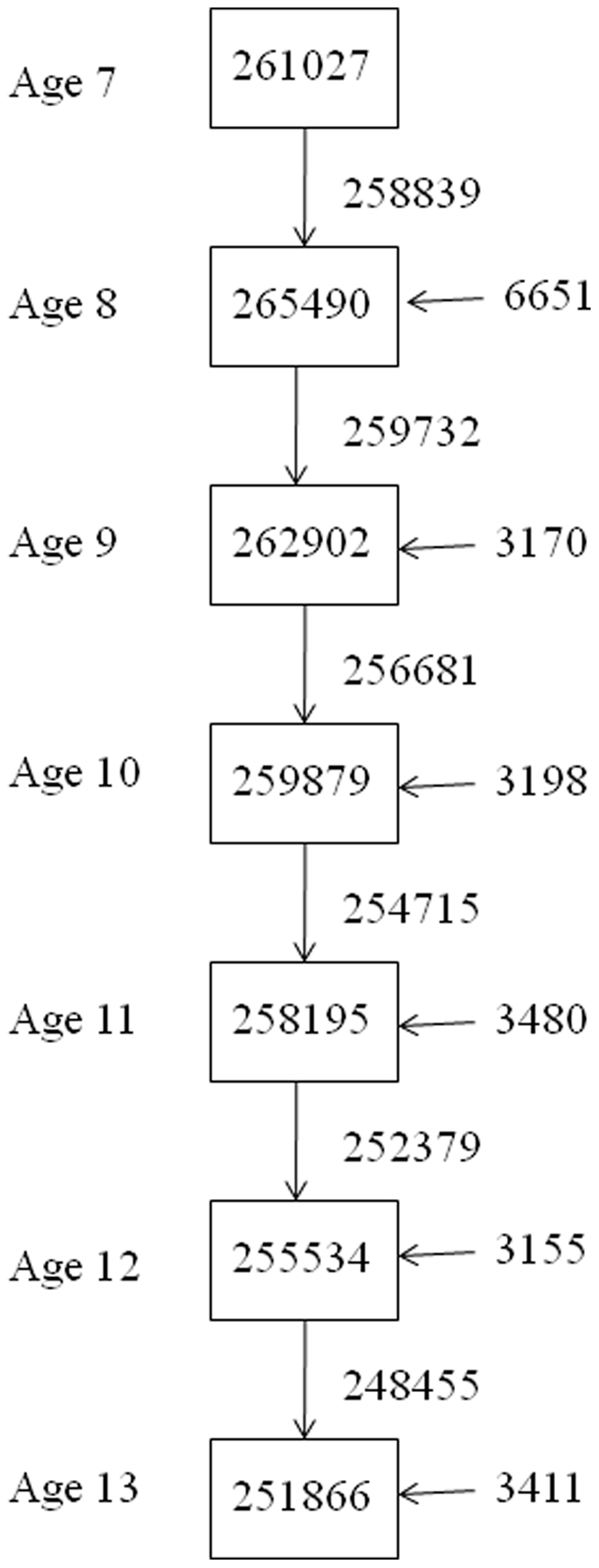
Number of children at each age, number of children measured also at the subsequent age and new children at the subsequent age.

The data are from the Copenhagen School Health Records Register described in detail elsewhere [11]. In short, the study cohort includes all children born from 1930 through 1976 and who attended school in the Copenhagen municipality. Physicians or nurses measured height without shoes during annual health examinations at public and private schools. Height from 7 to 13 years of age was computerized, and since the school year 1943 (birth year 1936) information on birth weight was available as provided by parental report at the first school examination. The number of participants varied from 265,490 at 8 years of age to 251,866 at 13 years of age, and the height was available at all ages for 232,063 children (82% of all participants) (Figure 1).

Follow-up data were obtained from the National Cause of Death Register [12] and the National Hospital Discharge Register [13], and were linked to the anthropometric data using unique personal identification numbers. The coverage of these registers is very high with virtually every event recorded. CHD diagnoses were classified according to the International Classification of Diseases Eighth Revision (ICD-8 codes 410–414) before 1994 and the Tenth Revision (ICD-10 codes I20–I25) thereafter. In the Hospital Discharge Register, myocardial infarctions are recorded with a high degree of validity [14], but in the National Cause of Death Register, cardiovascular disease diagnoses are less accurate [12]. Follow-up of the subjects began at 25 years of age or in 1977 when the Hospital Discharge Register was established. Age 25 was chosen as the earliest entry age since CHD at younger age may be because of congenital defects. The subjects were followed-up until a fatal or non-fatal CHD event, death from other causes, emigration or to the date of the latest register update on December 31^st^, 2008, whichever came first. During the follow-up period, 25,214 new CHD events occurred, 67% of them in men, including 6623 fatal events. From all CHD cases, 41% were because of acute myocardial infarction (ICD-8 code 410 and ICD-10 code I21) and 39% because of angina pectoris (413 and I20, respectively). The median follow-up time was 28.9 years. Of all CHD cases, 58% (14,665 cases) occurred before 60 years of age, and these were classified as early CHD cases in this study.

To allow the comparison of risks associated with height among boys and girls, at different ages and by year of birth, age-, sex- and birth cohort-specific standardized height values called height z-scores were computed, where one standard deviation of the sex, birth-cohort and age-specific distribution of height is the unit. Since standard deviations of height were very similar across birth cohorts in all ages and in boys and girls (Table S1), one unit change of z-score can be interpreted as a change of one standard deviation in height. The height z-scores were interpolated to exact ages if two measurements were available or extrapolated to exact ages if only one measurement was available. The birth weight z-scores were calculated based on an internal reference from the birth years of 1955 to 1960. The birth cohorts were divided into five intervals (1930 to 1935, 1936 to 1939, 1940 to 1945, 1946 to 1952, and 1953 to 1976) [15].

Hazard ratios (HR) and 95% confidence intervals (CI) for the risk of all CHD events (fatal or non-fatal) were first computed for height z-scores at each age from 7 to 13 years using Cox proportional hazards regression with age as the underlying time scale. Thus the Cox model estimates relative hazards for covariates, such as height z-score in these analyses, across the range of ages. Next we analyzed the association between growth in height and the risk of CHD by calculating differences in height z-scores between subsequent two year intervals and computed the HRs for this change with and without adjusting the baseline height z-score. Further we adjusted the results for baseline body mass index (BMI). Since the z-scores were either interpolated or extrapolated to exact ages, measures of adjacent ages are not necessarily based on separate measures, thus precluding the analysis of yearly changes. These analyses were also repeated using only fatal CHD cases and CHD cases before 60 years of age. Finally we analyzed potential non-linearity between growth in height and CHD risk; after dividing z-scores of birth weight and height at 7 and 13 years of age into quartiles, we estimated the HRs for different combinations of these quartiles. We tested the interactions between birth weight and the height quartiles using them as categorized variables. This was done by comparing χ^2^-statistics according to degrees of freedom between models having all main and interaction effects with models that only included main effects.

Year of birth was included as a continuous variable in all models to account for any linear association between year of birth and CHD risk within the five birth cohorts. Birth cohort was also used as a stratum variable in the models thus allowing different baseline hazards for each of them. Because growth is different in boys and girls, separate analyses were conducted by sex. The exception was the analyses of growth between birth weight and height at 7 and 13 years of age since the number of cases in some categories was too small for sex-specific analyses; in these analyses sex was used as a stratum variable. Stata statistical software, version 10.1, was used to conduct the analyses.

Since the study is entirely based on register data, only an approval by the Danish Data Protection Agency was required, and it was obtained.

## Results

Boys were nearly 1 cm taller than girls until age 11 at which age the difference in height disappeared and thereafter girls were slightly taller (Table 1). Growth was most rapid between 11 and 12 years of age in girls (6.2 cm) and between 12 and 13 years of age in boys (5.8 cm). From 10 to 12 years of age, the standard deviation was larger in girls than in boys whereas the opposite was true at age 13. A clear increase in the mean height across the birth years was observed (Table S1). When calculated using year of birth as a continuous measure, mean height increased 1.1 cm per decade in boys and 1.0 cm per decade in girls at the age of 7, and the mean increase was 1.5 cm per decade in both boys and girls at the age of 13.

**Table 1:** Number of participants, means and standard deviations (SD) of birth weight and of height from 7 to 13 years of age.

	Boys			Girls		
	N	mean	SD	N	mean	SD
Birth weight (kg)	104,494	3.42	0.614	108,875	3.29	0.599
Height at age 7 (cm)	131,910	123.4	5.43	129,117	122.5	5.40
Height at age 8 (cm)	134,143	127.6	5.53	131,347	126.6	5.52
Height at age 9 (cm)	132,679	132.9	5.82	130,223	131.9	5.83
Height at age 10 (cm)	130,943	138.0	6.07	128,936	137.2	6.23
Height at age 11 (cm)	129,994	142.8	6.38	128,201	142.8	6.83
Height at age 12 (cm)	128,478	147.8	6.94	127,056	149.0	7.35
Height at age 13 (cm)	126,297	153.6	7.88	125,569	154.9	7.20

Height was inversely associated with CHD risk at all ages in boys and girls (Table 2). When adjusted only for birth year (Model 1), the association was strongest at 7 years of age (HR = 0.91 in boys and 0.88 in girls) and weakened thereafter, with HR values approaching 1, but it still remained present at 13 years of age (HR = 0.95 and 0.91, respectively). When only CHD cases before 60 years of age or fatal CHD cases were analyzed, the age pattern was similar but the risks were systematically stronger than for all CHD cases in boys and girls (Table S2). We then adjusted the results additionally for birth weight in the sub-cohort where this information was available (Model 2) and found that HR values were very similar to those without this adjustment. Finally we adjusted the results for BMI (Model 3) and found that the association became stronger; this effect was stronger at older ages and thus the age pattern was weaker in the BMI adjusted results, especially in boys.

**Table 2:** Hazard ratios (HR) with 95% confidence intervals (CI) for CHD incidence per 1 unit increase in z-scores of height from 7 to 13 years of age.

	Boys						Girls					
	Model 1		Model 2		Model 3		Model 1		Model 2		Model 3	
	HR	95% CI	HR	95% CI	HR	95% CI	HR	95% CI	HR	95% CI	HR	95% CI
Age 7	0.91	0.90–0.92	0.91	0.89–0.93	0.90	0.89–0.91	0.88	0.86–0.90	0.88	0.85–0.90	0.87	0.85–0.89
Age 8	0.91	0.90–0.92	0.91	0.89–0.93	0.89	0.88–0.91	0.89	0.87–0.91	0.87	0.85–0.90	0.88	0.86–0.90
Age 9	0.92	0.90–0.93	0.92	0.90–0.94	0.89	0.88–0.91	0.89	0.87–0.91	0.88	0.85–0.91	0.88	0.86–0.90
Age 10	0.92	0.91–0.93	0.92	0.90–0.94	0.89	0.88–0.91	0.90	0.88–0.92	0.88	0.86–0.91	0.88	0.86–0.90
Age 11	0.93	0.91–0.94	0.93	0.91–0.95	0.90	0.88–0.91	0.91	0.89–0.93	0.90	0.87–0.93	0.89	0.87–0.91
Age 12	0.93	0.92–0.95	0.94	0.92–0.96	0.90	0.88–0.91	0.92	0.90–0.94	0.91	0.88–0.93	0.89	0.87–0.91
Age 13	0.95	0.94–0.97	0.96	0.94–0.98	0.91	0.89–0.92	0.91	0.89–0.93	0.90	0.87–0.92	0.88	0.86–0.90

Model 1 = adjusted for birth cohort; Model 2 = adjusted for birth cohort and birth weight in a sub-cohort; Model 3 = adjusted for birth cohort and BMI at baseline.

Rapid growth in height between 7 and 9 years of age, measured as change in relative height between these two ages, was associated with an increased CHD risk in boys and girls (HR = 1.11 and 1.17 for 1-unit change in height z-scores, respectively) (Table 3). In boys, the CHD risk increased steadily and was strongest for growth between 11 and 13 years of age (HR = 1.28). In girls this association was strongest between 9 and 11 years of age (HR = 1.22) and then disappeared when adjusted only for birth year (Model 1). When only CHD cases before 60 years of age or fatal CHD cases were analyzed, the strongest associations were also seen between 11 and 13 years of age in boys and between 9 and 11 years of age in girls, but in boys the associations were stronger than for all CHD cases (Table S3). Adjustment for height (Model 2) and BMI (Model 3) at baseline attenuated these associations, but growth from 9 to 11 and 11 to 13 years of age in boys and from 11 to 13 years of age in girls was still statistically significant.

**Table 3:** Hazard ratios (HR) with 95% confidence intervals (CI) of CHD incidence for 1 unit change in z-scores between 7 and 13 years of age.

	Boys						Girls					
	Model 1		Model 2		Model 3		Model 1		Model 2		Model 3	
	HR	95% CI	HR	95% CI	HR	95% CI	HR	95% CI	HR	95% CI	HR	95% CI
Age 7 to Age 9	1.11	1.04–1.18	1.05	0.98–1.12	1.02	0.96–1.09	1.17	1.08–1.28	1.09	1.00–1.19	1.08	0.99–1.17
Age 9 to Age 11	1.23	1.15–1.32	1.18	1.10–1.27	1.13	1.05–1.21	1.22	1.14–1.31	1.16	1.08–1.25	1.14	1.06–1.22
Age 11 to Age 13	1.28	1.22–1.33	1.24	1.19–1.30	1.13	1.05–1.21	1.01	0.95–1.08	0.97	0.91–1.04	0.99	0.92–1.05

Model 1: Adjusted for birth cohort; Model 2: Adjusted for birth cohort and height z-score at baseline; Model 3: Adjusted for birth cohort and height z-score and BMI at baseline.

**Figure 2:**
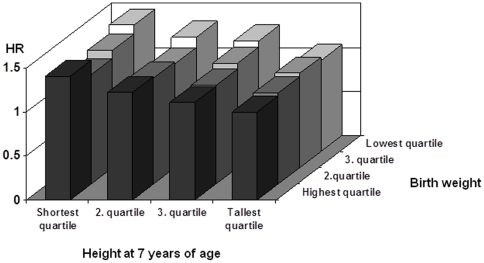
Hazard ratios of CHD risk by quartiles of height z-score at age 7 and birth weight z-score in the pooled data of men and women.

Next, we analyzed whether the association between height z-score at age 7 and CHD risk varied according to birth weight z-score. The risk of CHD steadily increased from the tallest to the shortest quartile of height in all quartiles of birth weight (Figure 2). There was no statistically significant (p = 0.26) interaction among height z-score quartiles at age 7 and birth weight z-score quartiles on the risk of CHD.

**Figure 3:**
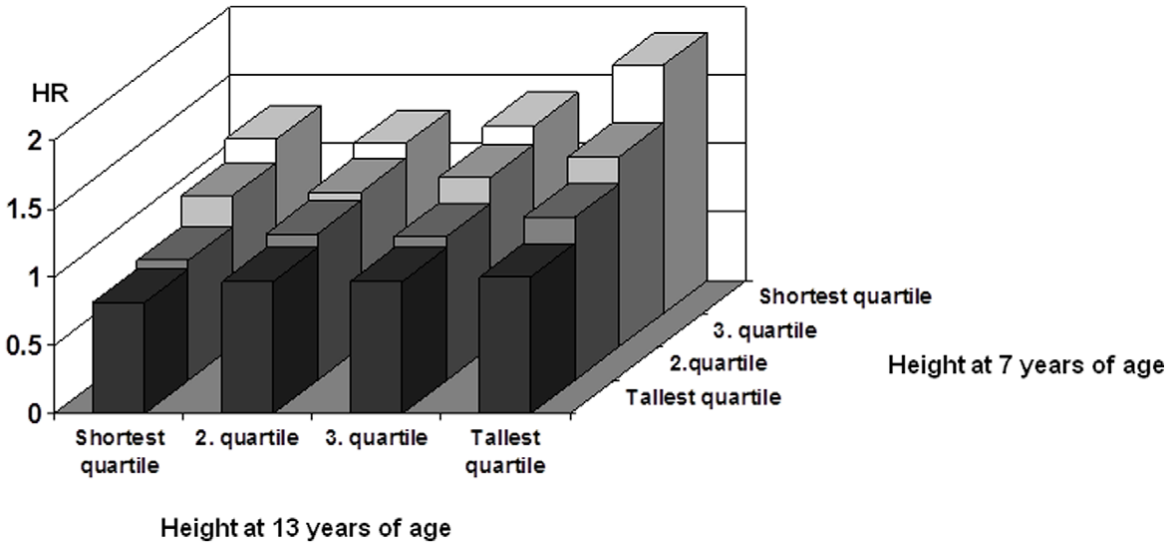
Hazard ratios of CHD risk by quartiles of height z-score at age 7 and 13 in the pooled data of men and women.

Next we conducted the same type of analysis between height z-scores at ages of 7 and 13 (Figure 3). This analysis showed that the highest risk was in those participants who were in the shortest height z-score quartile at 7 years of age and in the tallest quartile at 13 years of age (HR = 1.82, 95% CI 1.36–2.42), but otherwise no evidence of an interactive effect was seen. There was not a statistically significant overall interaction effect among height z-score quartiles at 7 and 13 years of age (p = 0.07).

We finally tested the heterogeneity of the associations of height and growth with CHD incidence over the birth cohorts. Because we found that the HRs were slightly stronger for CHD cases before 60 years of age than for all CHD cases, we conducted these analyses only for CHD cases before 60 years of age to account for differences in follow-up time among the birth cohorts. We found that the interactions between birth cohort and height at different ages were not statistically significant (Table S4). Growth from 7 to 9 years of age showed a statistically significant interaction with birth cohort in boys (p = 0.002) which was because of relatively high HRs in the cohort born 1940 to 1945 (HR 1.48 95% CI 1.27–1.72). For growth from ages 11 to 13 years, a relatively high HR in the cohort born 1936 to 1939 was found (HR 1.56 95% CI 1.35–1.79), but the overall interaction was not statistically significant (p = 0.158). Otherwise, the HRs were very similar in all birth cohorts and the interactions between birth cohort and growth were not statistically significant (Table S5). Thus these results suggested that the associations or height and growth with CHD incidence later in life are largely similar across the birth cohorts.

## Discussion

In this large longitudinal cohort we found that shorter stature in children from age 7 to 13 years was associated with an increased risk of CHD in adulthood, and these associations were not modified by birth weight. Height z-scores predicted the later risk of CHD with approximately the same effect size as did BMI z-scores in these same data [15], but the direction of the effect and changes in strength of the associations across the age groups were opposite. The associations between height and CHD showed a clear age pattern: they were strongest at the age of 7 and decreased until the age of 13 in both boys and girls. This pattern was partly because of the effect of BMI; when the results were adjusted for BMI the association between height and further CHD incidence become stronger because of correlation between height and BMI (r from 0.18 to 0.30), and this change was more visible at older ages. These findings strongly suggest that further investigations of the underlying biological processes that the growth-CHD association is driven by are warranted. The associations were slightly stronger for CHD cases before 60 years of age and the fatal events than for all fatal and non-fatal events together. The associations are measured on the multiplicative hazard ratio scale, which is sensitive to the baseline hazards - the lower it is the stronger the association for a given excess incidence rate. Whether the growth patterns are truly differently associated with the various manifestations of CHD, ranging from first diagnosis of angina pectoris through immediate death, is an open question beyond the scope of our present study.

The association between short adult stature and increased CHD risk has been known since the 1980s [16] and has been replicated in numerous studies since then [4]. However studies on the association between height in childhood and later CHD risk are sparse. In the Hertfordshire study it was reported for the first time that low birth weight in men and women as well as low weight at one year of age in males was associated with a higher risk of cardiovascular diseases [17], and thereafter it has been found in several studies that low birth weight is associated with increased risk of cardiovascular disease [3] as well as its risk factors [2]. In another UK cohort of children 2 to 14 years of age at baseline, leg-length and height were inversely associated with CHD mortality in adulthood [18].

Our results show that it is necessary to disentangle the effects of a generally slow growth trajectory leading to short stature in adults and a fast growth trajectory during late childhood; both are associated with increased risk of later CHD, and are therefore masking the effects of each other in the final stature. Thus the decreasing strength of the association between height and the risk of CHD across childhood probably reflects a mixture of opposing effects. The inverse association between childhood height and risk of CHD in adulthood may be diluted by the opposite effect of rapid growth that increases the CHD risk. The tallest boys and girls at 13 years of age thus appear to be a heterogeneous group with regard to CHD risk. Those who were taller-than-average at 7 years of age and remained tall had the lowest CHD risk. In contrast, those who were shorter-than-average at 7 years of age and then grew and became taller-than-average at 13 years of age had an increased CHD risk, which even exceeded the risk of the children who remained shorter-than-average between 7 and 13 years of age. Rapid growth was associated with higher BMI, but the association between rapid growth and CHD risk remained even when adjusting the results for baseline BMI.

Interestingly, rapid growth between 11 and 13 years of age in boys and between 9 and 11 years of age in girls showed the strongest association with increased CHD risk. These ages closely coincide with the timing of puberty in the Danish population both measured as sexual maturation [19] and as the onset of pubertal growth spurt [20]. In this same cohort, the average age at the onset of pubertal growth spurt was 12.2 years in boys born from 1930 to 1940 and decreased to 11.8 years in those born from 1965 to 1970 whereas in girls the ages were 10.4 and 10.2 years, respectively [20]. A Finnish study has also shown that rapid growth during these periods correlates closely with early sexual maturation [21]. Thus it is likely that children in our cohort who experienced rapid growth at these ages also had an early onset of puberty. This is consistent with the finding of high risk of CHD in the boys and girls who were in the shortest height quartile at 7 years of age but in the tallest quartile at 13 years of age. Previous studies have shown that early onset of puberty is associated with the emergence of several risk factors for CHD including hypertension [7], obesity [22], adverse blood lipid profile [8] and high levels of insulin [23] in adulthood. Our study thus suggests that the growth rate during the age of pubertal development is associated with increased risk of CHD in adulthood, and this association is very similar in men and women despite their different timing of puberty.

It is noteworthy that rapid growth and increased CHD risk was seen already between 7 and 9 years of age in both boys and girls thus well preceding the onset of puberty. A Finnish study found that participants diagnosed with hypertension at 63 years of age had a lower birth weight and had showed catch-up growth in height and weight until 11 years of age when compared to normotensive men and women. The individuals, however, who were newly diagnosed as hypertensive in the clinical examination showed a different growth pattern since they had low birth weight, but did not show catch-up growth [24]. In this same Finnish cohort it was also found that in females short length at birth was associated with higher risk of CHD in adulthood, and this association was strongest in those women who had experiences catch-up growth until 7 years of age [25].

We found no evidence supporting that only children with a low birth weight combined with later rapid growth in height have a higher CHD risk; the association between height in childhood and later CHD risk was largely independent of birth weight. This result is consistent with a previous Finnish study, which found no evidence of catch-up growth from 2 to 10 years of age in the persons who had had coronary event in adulthood [6]. The results rather suggest that the height growth velocity in childhood carries a risk for CHD independent of birth weight. These associations likely reflect the combined effects of nutritional, environmental, genetic and hormonal factors, such as the secretion of insulin-like growth factor, on growth [26]. For example, it has been found that leg length in childhood is more strongly associated with CHD risk than trunk length, which may be because leg length is a more sensitive indicator of environmental factors than trunk length [18]. There is also evidence based mainly on Finnish studies that growth in utero and infancy may be associated with the proportion of lean body mass and lipid metabolism in adulthood [10].

The association between short stature-to-age in childhood and later CHD risk may at least partly reflect the role of socio-economic factors, since it is well known that low socioeconomic position in childhood is associated with shorter adult stature [27] and that clear socioeconomic differences in CHD risk are present in Northern-Europe and the USA [28]. We did not have access to information on parental socio-economic position, so we could not directly test this hypothesis. However, a previous Nordic study found an association between adult stature and CHD risk within discordant twin pairs suggesting that socio-economic background cannot explain the association [29]. Further, we found that the association between stature and CHD was similar across birth cohorts despite a clear secular trend in height and improvements in the standard of living in Danish society across the birth cohorts. Taken together, these results suggest that the association between height and CHD risk is not strongly modified by socioeconomic position, and we neither find reasons to suspect that rapid growth in childhood is associated with low socio-economic position. Moreover, the study design and completeness of follow-up make it very unlikely that any socioeconomic selection bias exists.

The biological pathways underlying the association of CHD incidence with growth are still largely unknown. In addition to the above mentioned hypotheses on the role of nutritional, metabolic, hormonal and socioeconomic factors as mediators of this association, there are also other possible explanations. It has been suggested that short stature might be a CHD risk factor itself since it correlates with narrow coronary arteries, which may further predispose to CHD [30]. However our results did not give support for this hypothesis since we found that the association between height at 13 years of age and further CHD incidence depended on the growth velocity from 7 to 13 years of age. Further, there may be other mechanisms that are currently unknown. To answer these types of questions data sets containing information on growth in childhood and detailed metabolic as well as other biological measurements in adulthood are required. Future research can be undertaken to investigate these potentially mediating pathways in depth by using information from detailed examinations in much smaller subgroups of individuals selected on the basis of various growth patterns in childhood.

Our study has several strengths, but also limitations. We used a very large population-based cohort with annual height measures from 7 through 13 years of age. The health examinations were performed at all public and private schools and thus cover virtually every schoolchild in the Copenhagen municipality born from 1930 to 1976. Our follow-up data cover also all study participants because of universal health care system in Denmark. Thus, it is very unlikely that any selection bias exists at baseline or during follow-up. In the present cohort, we lack information on conventional risk factors for CHD, and it will be a task for future studies to elaborate which metabolic or other biological factors may underlie the observed associations between childhood growth and further CHD risk. Further since we do not have any measurements of sexual maturation, such as age at the onset of menarche, and we cannot determinate peak growth velocity for late mature children our results on the associations between timing of puberty and further CHD incidence can be regarded only tentative. It is also noteworthy that our study population represents an ethnically homogeneous Caucasian population with tall average stature [31]. There is little research on the associations between childhood height and CHD risk in other ethnic groups. However, for adult stature the association was very similar in a study of Korean men [32] to that found in studies conducted in adult Caucasian populations [4] suggesting that the association between growth and CHD risk would be present also in other ethnic groups as well.

To summarize, we found that shorter stature in childhood was associated with increased CHD risk in adulthood, that it grew weaker with age, and that the association was independent of birth weight. Rapid pre-pubertal growth, but especially rapid growth close to puberty, was associated with increased CHD risk, demonstrating the importance of physical development of the child for the risk. Physical development in childhood thus captures important information on the childhood environment, such as socio-economic factors and nutrition, which are important for further cardio-vascular health. Thus our results support the importance of optimal living conditions in childhood for a future healthy life. Unraveling the biological pathways of this association and the exploration of the utility of the association in prevention of CHD are tasks for future research.

## Supporting Information

Table S1 Number of participants, means (cm) and standard deviations (SD) of height from 7 to 13 years of age by five birth cohorts.
(DOC)

Table S2 Hazard ratios (HRs) with 95% confidence intervals (CI) for early and fatal CHD incidence cases per 1 unit increase in z-scores of height from 7 to 13 years of age.
(DOC)

Table S3 Hazard ratios (HRs) with 95% confidence intervals (CI) of early and fatal CHD incidence cases for 1 unit change in z-scores between 7 and 13 years of age.
(DOC)

Table S4 Hazard ratios (HR) with 95% confidence intervals (CI) for CHD incidence before 60 years of age per 1 unit increase in z-scores of height from 7 to 13 years of age by birth cohort.
(DOC)

Table S5 Hazard ratios (HR) with 95% confidence intervals (CI) for CHD incidence before 60 years of age for 1 unit change in z-scores between 7 and 13 years of age by birth cohort.
(DOC)

## References

[1] Singhal A, Lucas A (2004). Early origins of cardiovascular disease: is there a unifying hypothesis?. Lancet.

[2] Gamborg M, Byberg L, Rasmussen F, Andersen PK, Baker JL (2007). Birth weight and systolic blood pressure in adolescence and adulthood: meta-regression analysis of sex- and age-specific results from 20 Nordic studies.. Am J Epidemiol.

[3] Risnes KR, Vatten LJ, Baker JL, Jameson K, Sovio U (2011). Birthweight and mortality in adulthood: a systematic review and meta-analysis.. Int J Epidemiol.

[4] Paajanen TA, Oksala NK, Kuukasjärvi P, Karhunen PJ (2010). Short stature is associated with coronary heart disease: a systematic review of the literature and a meta-analysis.. Eur Heart J.

[5] Sørensen HT, Sabroe S, Rothman KJ, Gillman M, Steffensen FH (1999). Birth weight and length as predictors for adult height.. Am J Epidemiol.

[6] Barker DJ, Osmond C, Forsen TJ, Kajantie E, Eriksson JG (2005). Trajectories of growth among children who have coronary events as adults.. N Engl J Med.

[7] Hardy R, Kuh D, Whincup PH, Wadsworth ME (2006). Age at puberty and adult blood pressure and body size in a British birth cohort study.. J Hypertens.

[8] Feng Y, Hong X, Wilker E, Li Z, Zhang W (2008). Effects of age at menarche, reproductive years, and menopause on metabolic risk factors for cardiovascular diseases.. Atherosclerosis.

[9] Silventoinen K, Haukka J, Dunkel L, Tynelius P, Rasmussen F (2008). Genetics of pubertal timing and its associations with relative weight in childhood and adult height: the Swedish Young Male Twins Study.. Pediatrics.

[10] Barker DJ, Osmond C, Kajantie E, Eriksson JG (2009). Growth and chronic disease: findings in the Helsinki Birth Cohort.. Ann Hum Biol.

[11] Baker JL, Olsen LW, Andersen I, Pearson S, Hansen B (2009). Cohort Profile: The Copenhagen School Health Records Register.. Int J Epidemiol.

[12] Juel K, Helweg-Larsen K (1999). The Danish registers of causes of death.. Dan Med Bull.

[13] Andersen TF, Madsen M, Jorgensen J, Mellemkjoer L, Olsen JH (1999). The Danish National Hospital Register. A valuable source of data for modern health sciences.. Dan Med Bull.

[14] Madsen M, Davidsen M, Rasmussen S, Abildstrom SZ, Osler M (2003). The validity of the diagnosis of acute myocardial infarction in routine statistics: a comparison of mortality and hospital discharge data with the Danish MONICA registry.. J Clin Epidemiol.

[15] Baker JL, Olsen LW, Sørensen TIA (2007). Childhood body-mass index and the risk of coronary heart disease in adulthood.. NEJM.

[16] Waaler HT (1984). Height, weight and mortality. The Norwegian experience. Acta Med Scand.

[17] Osmond C, Barker DJ, Winter PD, Fall CH, Simmonds SJ (1993). Early growth and death from cardiovascular disease in women.. BMJ.

[18] Gunnell DJ, Davey Smith G, Frankel S, Nanchahal K, Braddon FE (1998). Childhood leg length and adult mortality: follow up of the Carnegie (Boyd Orr) Survey of Diet and Health in Pre-war Britain.. J Epidemiol Community Health.

[19] Juul A, Teilmann G, Scheike T, Hertel NT, Holm K (2006). Pubertal development in Danish children: comparison of recent European and US data.. Int J Androl.

[20] Aksglaede L, Olsen LW, Sørensen TIA, Juul A (2008). Forty years trends in timing of pubertal growth spurt in 157,000 Danish school children.. PLoS One.

[21] Wehkalampi K, Silventoinen K, Kaprio J, Dick DM, Rose RJ (2008). Genetic and environmental influences on pubertal timing assessed by height growth.. Am J Hum Biol.

[22] Kindblom JM, Lorentzon M, Norjavaara E, Lonn L, Brandberg J (2006). Pubertal timing is an independent predictor of central adiposity in young adult males: the Gothenburg osteoporosis and obesity determinants study.. Diabetes.

[23] Frontini MG, Srinivasan SR, Berenson GS (2003). Longitudinal changes in risk variables underlying metabolic Syndrome X from childhood to young adulthood in female subjects with a history of early menarche: the Bogalusa Heart Study.. Int J Obes.

[24] Eriksson JG, Forsen TJ, Kajantie E, Osmond C, Barker DJ (2007). Childhood growth and hypertension in later life.. Hypertension.

[25] Forsen T, Eriksson JG, Tuomilehto J, Osmond C, Barker DJ (1999). Growth in utero and during childhood among women who develop coronary heart disease: longitudinal study.. BMJ.

[26] Sandhu J, Davey Smith G, Holly J, Cole TJ, Ben-Shlomo Y (2006). Timing of puberty determines serum insulin-like growth factor-I in late adulthood.. J Clin Endocrinol Metab.

[27] Silventoinen K (2003). Determinants of variation in adult body height.. J Biosoc Sci.

[28] Mackenbach JP, Cavelaars AE, Kunst AE, Groenhof F (2000). Socioeconomic inequalities in cardiovascular disease mortality; an international study.. Eur Heart J.

[29] Silventoinen K, Zdravkovic S, Skytthe A, McCarron P, Herskind AM (2006). Association between height and coronary heart disease mortality: a prospective study of 35,000 twin pairs.. Am J Epidemiol.

[30] Nwasokwa ON, Weiss M, Gladstone C, Bodenheimer MM (1997). Higher prevalence and greater severity of coronary disease in short versus tall men referred for coronary arteriography.. Am Heart J.

[31] Eveleth PB, Tanner JM (2003). Worldwide variation in human growth. 2nd ed.

[32] Song YM, Smith GD, Sung J (2003). Adult height and cause-specific mortality: a large prospective study of South Korean men.. Am J Epidemiol.

