# Supplementary material for: Growth in Height in Childhood and Risk of Coronary Heart Disease in Adult Men and Women
Source: PLoS One. 2012 Jan 24;7(1):e30476. doi: 10.1371/journal.pone.0030476 (PMC3265486; doi:10.1371/journal.pone.0030476)
Supplement: Table S1 — Number of participants, means (cm) and standard deviations (SD) of height from 7 to 13 years of age by five birth cohorts. (DOC) [file pone.0030476.s001.doc]

Table S1. Number of participants, means (cm) and standard deviations (SD) of height from 7 to 13 years of age by five birth cohorts.

|  | Boys | | | Girls | | |
| --- | --- | --- | --- | --- | --- | --- |
|  | N | mean | SD | N | mean | SD |
| Cohort 1930 to 1935 | | | | | | |
| Age 7 | 15596 | 120.8 | 5.16 | 15213 | 120.1 | 5.21 |
| Age 8 | 15841 | 125.5 | 5.39 | 15481 | 124.7 | 5.42 |
| Age 9 | 15768 | 130.6 | 5.60 | 15481 | 129.8 | 5.59 |
| Age 10 | 15707 | 135.5 | 5.77 | 15456 | 134.7 | 5.87 |
| Age 11 | 15641 | 140.1 | 6.01 | 15490 | 139.9 | 6.36 |
| Age 12 | 15368 | 144.7 | 6.43 | 15419 | 145.5 | 6.95 |
| Age 13 | 14733 | 150.0 | 7.18 | 15180 | 151.5 | 7.02 |
| Cohort 1936 to 1939 | | | | | | |
| Age 7 | 13592 | 121.5 | 5.19 | 15064 | 120.6 | 5.28 |
| Age 8 | 13938 | 126.2 | 5.40 | 13363 | 125.3 | 5.46 |
| Age 9 | 14038 | 131.5 | 5.57 | 13540 | 130.4 | 5.59 |
| Age 10 | 14122 | 136.4 | 5.79 | 13700 | 135.4 | 5.88 |
| Age 11 | 14133 | 141.2 | 6.06 | 13850 | 141.0 | 6.50 |
| Age 12 | 14092 | 146.2 | 6.51 | 13941 | 147.2 | 7.01 |
| Age 13 | 13940 | 151.9 | 7.35 | 13853 | 153.3 | 7.00 |
| Cohort 1940 to 1945 | | | | | | |
| Age 7 | 26120 | 122.9 | 5.13 | 25618 | 122.0 | 5.14 |
| Age 8 | 26506 | 127.4 | 5.32 | 26109 | 126.3 | 5.34 |
| Age 9 | 26496 | 132.8 | 5.57 | 26130 | 131.6 | 5.60 |
| Age 10 | 26407 | 137.8 | 5.79 | 26100 | 136.8 | 5.96 |
| Age 11 | 26435 | 142.6 | 6.09 | 26061 | 142.4 | 6.54 |
| Age 12 | 26389 | 147.5 | 6.61 | 26017 | 148.6 | 7.05 |
| Age 13 | 26234 | 153.3 | 7.49 | 25955 | 154.6 | 6.90 |
| Cohort 1946 to 1952 | | | | | | |
| Age 7 | 27222 | 124.0 | 5.30 | 26874 | 122.9 | 5.27 |
| Age 8 | 27675 | 127.6 | 5.37 | 27260 | 126.6 | 5.36 |
| Age 9 | 27638 | 133.0 | 5.64 | 27229 | 132.0 | 5.66 |
| Age 10 | 27617 | 138.2 | 5.89 | 27222 | 137.4 | 6.04 |
| Age 11 | 27591 | 143.0 | 6.19 | 27202 | 143.1 | 6.63 |
| Age 12 | 27540 | 148.0 | 6.74 | 27161 | 149.3 | 7.10 |
| Age 13 | 27453 | 153.9 | 7.67 | 27137 | 155.3 | 6.94 |
| Cohort 1953 to 1976 | | | | | | |
| Age 7 | 49380 | 124.7 | 5.34 | 48425 | 123.7 | 5.29 |
| Age 8 | 50183 | 128.8 | 5.50 | 49134 | 127.8 | 5.48 |
| Age 9 | 48739 | 134.2 | 5.86 | 47843 | 133.2 | 5.87 |
| Age 10 | 47090 | 139.3 | 6.14 | 46458 | 138.7 | 6.28 |
| Age 11 | 46194 | 144.2 | 6.48 | 45598 | 144.4 | 6.86 |
| Age 12 | 45089 | 149.2 | 7.11 | 44518 | 150.7 | 7.34 |
| Age 13 | 43937 | 155.3 | 8.09 | 43444 | 156.7 | 7.12 |
