# Supplementary material for: Growth in Height in Childhood and Risk of Coronary Heart Disease in Adult Men and Women
Source: PLoS One. 2012 Jan 24;7(1):e30476. doi: 10.1371/journal.pone.0030476 (PMC3265486; doi:10.1371/journal.pone.0030476)
Supplement: Table S2 — Hazard ratios (HRs) with 95% confidence intervals (CI) for early and fatal CHD incidence cases per 1 unit increase in z-scores of height from 7 to 13 years of age. (DOC) [file pone.0030476.s002.doc]

Table S2. Hazard ratios (HRs) with 95% confidence intervals (CI) for early and fatal CHD incidence cases per 1 unit increase in z-scores of height from 7 to 13 years of age.

|  | Boys | | | | | | Girls | | | | | |
| --- | --- | --- | --- | --- | --- | --- | --- | --- | --- | --- | --- | --- |
|  | Model 1 | | Model 2 | | Model 3 | | Model 1 | | Model 2 | | Model 3 | |
|  | HR | 95% CI | HR | 95% CI | HR | 95% CI | HR | 95% CI | HR | 95% CI | HR | 95% CI |
| Early CHD cases (before 60 years of age) | | | | | | | | | | | | |
| Age 7 | 0.89 | 0.88-0.91 | 0.91 | 0.88-0.93 | 0.88 | 0.86-0.90 | 0.85 | 0.82-0.88 | 0.87 | 0.83-0.90 | 0.84 | 0.82-0.87 |
| Age 8 | 0.89 | 0.87-0.91 | 0.91 | 0.88-0.93 | 0.87 | 0.85-0.89 | 0.85 | 0.82-0.88 | 0.86 | 0.83-0.89 | 0.84 | 0.81-0.87 |
| Age 9 | 0.90 | 0.88-0.92 | 0.91 | 0.89-0.93 | 0.87 | 0.85-0.89 | 0.85 | 0.82-0.88 | 0.86 | 0.83-0.89 | 0.83 | 0.81-0.86 |
| Age 10 | 0.90 | 0.89-0.92 | 0.92 | 0.90-0.94 | 0.87 | 0.85-0.89 | 0.85 | 0.83-0.88 | 0.87 | 0.83-0.90 | 0.83 | 0.80-0.86 |
| Age 11 | 0.91 | 0.89-0.93 | 0.93 | 0.90-0.95 | 0.87 | 0.85-0.89 | 0.87 | 0.84-0.89 | 0.88 | 0.85-0.91 | 0.84 | 0.81-0.87 |
| Age 12 | 0.92 | 0.90-0.94 | 0.94 | 0.92-0.96 | 0.87 | 0.86-0.89 | 0.88 | 0.85-0.91 | 0.89 | 0.85-0.92 | 0.84 | 0.82-0.87 |
| Age 13 | 0.95 | 0.93-0.97 | 0.96 | 0.94-0.99 | 0.89 | 0.87-0.91 | 0.87 | 0.85-0.90 | 0.88 | 0.85-0.91 | 0.84 | 0.82-0.87 |
| Fatal CHD cases | | | | | | | | | | | | |
| Age 7 | 0.89 | 0.86-0.92 | 0.91 | 0.87-0.95 | 0.88 | 0.85-0.90 | 0.85 | 0.81-0.89 | 0.87 | 0.80-0.94 | 0.83 | 0.79-0.87 |
| Age 8 | 0.89 | 0.86-0.91 | 0.90 | 0.86-0.94 | 0.87 | 0.84-0.89 | 0.84 | 0.80-0.88 | 0.85 | 0.78-0.92 | 0.82 | 0.77-0.86 |
| Age 9 | 0.89 | 0.87-0.92 | 0.91 | 0.87-0.95 | 0.87 | 0.84-0.89 | 0.86 | 0.82-0.90 | 0.87 | 0.80-0.94 | 0.83 | 0.79-0.87 |
| Age 10 | 0.90 | 0.87-0.92 | 0.91 | 0.87-0.95 | 0.86 | 0.84-0.89 | 0.86 | 0.82-0.91 | 0.86 | 0.80-0.93 | 0.82 | 0.78-0.87 |
| Age 11 | 0.91 | 0.88-0.94 | 0.92 | 0.88-0.96 | 0.87 | 0.84-0.90 | 0.88 | 0.83-0.92 | 0.87 | 0.81-0.94 | 0.83 | 0.78-0.87 |
| Age 12 | 0.92 | 0.89-0.94 | 0.93 | 0.89-0.97 | 0.87 | 0.84-0.90 | 0.89 | 0.85-0.94 | 0.87 | 0.81-0.94 | 0.83 | 0.79-0.87 |
| Age 13 | 0.94 | 0.91-0.97 | 0.95 | 0.91-0.99 | 0.88 | 0.85-0.91 | 0.88 | 0.84-0.93 | 0.87 | 0.81-0.94 | 0.82 | 0.78-0.86 |

Model 1 = adjusted for birth cohort; Model 2 = adjusted for birth cohort and birth weight in a sub-cohort; Model 3 = adjusted for birth cohort and BMI at baseline
