# Supplementary material for: Growth in Height in Childhood and Risk of Coronary Heart Disease in Adult Men and Women
Source: PLoS One. 2012 Jan 24;7(1):e30476. doi: 10.1371/journal.pone.0030476 (PMC3265486; doi:10.1371/journal.pone.0030476)
Supplement: Table S3 — Hazard ratios (HRs) with 95% confidence intervals (CI) of early and fatal CHD incidence cases for 1 unit change in z-scores between 7 and 13 years of age. (DOC) [file pone.0030476.s003.doc]

Table S3. Hazard ratios (HRs) with 95% confidence intervals (CI) of early and fatal CHD incidence cases for 1 unit change in z-scores between 7 and 13 years of age.

|  |  | Boys | | | | | | Girls | | | | | |
| --- | --- | --- | --- | --- | --- | --- | --- | --- | --- | --- | --- | --- | --- |
|  | | Model 1 | | Model 2 | | Model 3 | | Model 1 | | Model 2 | | Model 3 | |
|  | | HR | 95% CI | HR | 95% CI | HR | 95% CI | HR | 95% CI | HR | 95% CI | HR | 95% CI |
| Early CHD cases (before 60 years of age) | | | | | | | | | | | | | |
| Age 7 to Age 9 | | 1.14 | 1.05-1.23 | 1.06 | 0.98-1.15 | 1.02 | 0.94-1.11 | 1.05 | 0.93-1.18 | 0.95 | 0.84-1.07 | 0.93 | 0.82-1.05 |
| Age 9 to Age 11 | | 1.32 | 1.21-1.44 | 1.26 | 1.15-1.38 | 1.19 | 1.08-1.30 | 1.27 | 1.16-1.40 | 1.19 | 1.08-1.31 | 1.16 | 1.05-1.28 |
| Age 11 to Age 13 | | 1.35 | 1.28-1.42 | 1.31 | 1.24-1.38 | 1.24 | 1.17-1.31 | 0.99 | 0.90-1.08 | 0.93 | 0.85-1.02 | 0.95 | 0.87-1.05 |
| Fatal CHD cases | | | | | | | | | | | | | |
| Age 7 to Age 9 | | 1.10 | 0.98-1.24 | 1.03 | 0.91-1.16 | 0.99 | 0.88-1.12 | 0.95 | 0.78-1.15 | 0.87 | 0.71-1.05 | 0.82 | 0.68-1.00 |
| Age 9 to Age 11 | | 1.26 | 1.10-1.44 | 1.19 | 1.04-1.36 | 1.12 | 1.98-1.28 | 1.22 | 1.04-1.43 | 1.14 | 0.97-1.34 | 1.08 | 0.91-1.26 |
| Age 11 to Age 13 | | 1.34 | 1.23-1.45 | 1.29 | 1.19-1.41 | 1.21 | 1.11-1.32 | 1.08 | 0.93-1.25 | 1.02 | 0.80-1.19 | 1.06 | 0.92-1.24 |

Model 1: Adjusted for birth cohort; Model 2: Adjusted for birth cohort and height z-score at baseline; Model 3: Adjusted for birth cohort and height z-score and BMI at baseline
