# Supplementary material for: Growth in Height in Childhood and Risk of Coronary Heart Disease in Adult Men and Women
Source: PLoS One. 2012 Jan 24;7(1):e30476. doi: 10.1371/journal.pone.0030476 (PMC3265486; doi:10.1371/journal.pone.0030476)
Supplement: Table S4 — Hazard ratios (HR) with 95% confidence intervals (CI) for CHD incidence before 60 years of age per 1 unit increase in z-scores of height from 7 to 13 years of age by birth cohort. (DOC) [file pone.0030476.s004.doc]

Table S4. Hazard ratios (HR) with 95% confidence intervals (CI) for CHD incidence before 60 years of age per 1 unit increase in z-scores of height from 7 to 13 years of age by birth cohort.

|  | Boys | | Girls | |
| --- | --- | --- | --- | --- |
|  | HR | 95% CI | HR | 95% CI |
| Age 7 | | | | |
| Cohort 1930 to 1935 | 0.88 | 0.84-0.93 | 0.84 | 0.77-0.91 |
| Cohort 1936 to 1939 | 0.87 | 0.82-0.92 | 0.84 | 0.77-0.92 |
| Cohort 1940 to 1945 | 0.88 | 0.84-0.91 | 0.85 | 0.80-0.91 |
| Cohort 1946 to 1952 | 0.92 | 0.88-0.95 | 0.88 | 0.83-0.93 |
| Cohort 1953 to 1976 | 0.91 | 0.86-0.96 | 0.82 | 0.76-0.88 |
| Cohort*height interaction | p=0.364 | | p=0.698 | |
| Age 8 | | | | |
| Cohort 1930 to 1935 | 0.89 | 0.85-0.93 | 0.85 | 0.78-0.92 |
| Cohort 1936 to 1939 | 0.86 | 0.82-0.91 | 0.85 | 0.78-0.93 |
| Cohort 1940 to 1945 | 0.88 | 0.85-0.92 | 0.85 | 0.80-0.90 |
| Cohort 1946 to 1952 | 0.91 | 0.87-0.94 | 0.87 | 0.82-0.92 |
| Cohort 1953 to 1976 | 0.91 | 0.86-0.96 | 0.82 | 0.76-0.88 |
| Cohort*height interaction | p=0.571 | | p=0.789 | |
| Age 9 | | | | |
| Cohort 1930 to 1935 | 0.90 | 0.85-0.94 | 0.83 | 0.76-0.90 |
| Cohort 1936 to 1939 | 0.86 | 0.82-0.91 | 0.86 | 0.78-0.94 |
| Cohort 1940 to 1945 | 0.90 | 0.86-0.93 | 0.86 | 0.81-0.91 |
| Cohort 1946 to 1952 | 0.91 | 0.87-0.94 | 0.87 | 0.82-0.92 |
| Cohort 1953 to 1976 | 0.91 | 0.86-0.96 | 0.82 | 0.76-0.88 |
| Cohort*height interaction | p=0.592 | | p=0.763 | |
| Age 10 | | | | |
| Cohort 1930 to 1935 | 0.90 | 0.85-0.94 | 0.84 | 0.77-0.91 |
| Cohort 1936 to 1939 | 0.87 | 0.83-0.92 | 0.84 | 0.77-0.92 |
| Cohort 1940 to 1945 | 0.90 | 0.87-0.94 | 0.86 | 0.81-0.92 |
| Cohort 1946 to 1952 | 0.91 | 0.88-0.95 | 0.88 | 0.83-0.93 |
| Cohort 1953 to 1976 | 0.92 | 0.87-0.97 | 0.82 | 0.76-0.88 |
| Cohort*height interaction | p=0.523 | | p=0.589 | |
| Age 11 | | | | |
| Cohort 1930 to 1935 | 0.90 | 0.86-0.95 | 0.86 | 0.79-0.94 |
| Cohort 1936 to 1939 | 0.87 | 0.83-0.92 | 0.86 | 0.79-0.94 |
| Cohort 1940 to 1945 | 0.91 | 0.88-0.95 | 0.88 | 0.83-0.93 |
| Cohort 1946 to 1952 | 0.92 | 0.89-0.96 | 0.89 | 0.84-0.94 |
| Cohort 1953 to 1976 | 0.93 | 0.88-0.98 | 0.83 | 0.77-0.89 |
| Cohort*height interaction | p=0.454 | | p=0.633 | |
| Age 12 | | | | |
| Cohort 1930 to 1935 | 0.91 | 0.86-0.95 | 0.90 | 0.83-0.97 |
| Cohort 1936 to 1939 | 0.88 | 0.84-0.93 | 0.86 | 0.79-0.94 |
| Cohort 1940 to 1945 | 0.92 | 0.89-0.96 | 0.87 | 0.82-0.93 |
| Cohort 1946 to 1952 | 0.94 | 0.90-0.98 | 0.90 | 0.85-0.96 |
| Cohort 1953 to 1976 | 0.94 | 0.89-0.99 | 0.84 | 0.78-0.91 |
| Cohort*height interaction | p=0.361 | | p=0.604 | |
| Age 13 | | | | |
| Cohort 1930 to 1935 | 0.93 | 0.89-0.98 | 0.89 | 0.82-0.96 |
| Cohort 1936 to 1939 | 0.92 | 0.87-0.96 | 0.87 | 0.80-0.95 |
| Cohort 1940 to 1945 | 0.95 | 0.91-0.99 | 0.86 | 0.81-0.92 |
| Cohort 1946 to 1952 | 0.96 | 0.92-1.00 | 0.90 | 0.85-0.95 |
| Cohort 1953 to 1976 | 0.97 | 0.92-1.02 | 0.85 | 0.79-0.91 |
| Cohort*height interaction | p=0.541 | | p=0.770 | |
