# Supplementary material for: Growth in Height in Childhood and Risk of Coronary Heart Disease in Adult Men and Women
Source: PLoS One. 2012 Jan 24;7(1):e30476. doi: 10.1371/journal.pone.0030476 (PMC3265486; doi:10.1371/journal.pone.0030476)
Supplement: Table S5 — Hazard ratios (HR) with 95% confidence intervals (CI) for CHD incidence before 60 years of age for 1 unit change in z-scores between 7 and 13 years of age by birth cohort. (DOC) [file pone.0030476.s005.doc]

Table S5. Hazard ratios (HR) with 95% confidence intervals (CI) for CHD incidence before 60 years of age for 1 unit change in z-scores between 7 and 13 years of age by birth cohort.

|  | Boys | | Girls | |
| --- | --- | --- | --- | --- |
|  | HR | 95% CI | HR | 95% CI |
| Age 7 to Age 9 | | | | |
| Cohort 1930 to 1935 | 1.10 | 0.91-1.33 | 0.91 | 0.66-1.25 |
| Cohort 1936 to 1939 | 1.15 | 0.94-1.41 | 1.02 | 0.75-1.38 |
| Cohort 1940 to 1945 | 1.48 | 1.27-1.72 | 1.18 | 0.94-1.49 |
| Cohort 1946 to 1952 | 0.96 | 0.80-1.14 | 1.01 | 0.80- 1.29 |
| Cohort 1953 to 1976 | 1.02 | 0.82-1.28 | 1.16 | 0.83-1.48 |
| Cohort*height interaction | p=0.002 | | p=0.698 | |
| Age 9 to Age 11 | | | | |
| Cohort 1930 to 1935 | 1.12 | 0.91-1.38 | 1.57 | 1.21-2.03 |
| Cohort 1936 to 1939 | 1.18 | 0.94-1.48 | 1.14 | 0.87-1.48 |
| Cohort 1940 to 1945 | 1.46 | 1.22-1.74 | 1.25 | 1.05-1.50 |
| Cohort 1946 to 1952 | 1.44 | 1.20-1.73 | 1.33 | 1.12-1.59 |
| Cohort 1953 to 1976 | 1.25 | 1.00-1.57 | 1.11 | 0.88-1.38 |
| Cohort*height interaction | p=0.154 | | p=0.249 | |
| Age 11 to Age 13 | | | | |
| Cohort 1930 to 1935 | 1.22 | 1.06-1.40 | 1.03 | 0.81-1.32 |
| Cohort 1936 to 1939 | 1.56 | 1.35-1.79 | 1.09 | 0.85-1.41 |
| Cohort 1940 to 1945 | 1.39 | 1.25-1.54 | 0.87 | 0.73-1.03 |
| Cohort 1946 to 1952 | 1.30 | 1.17-1.44 | 1.05 | 0.88-1.24 |
| Cohort 1953 to 1976 | 1.32 | 1.15-1.52 | 0.98 | 0.80-1.21 |
| Cohort*height interaction | p=0.158 | | p=0.473 | |
